# Comparative effects of arithmetic, speech, and motor dual-task walking on gait in stroke survivors: a cross-sectional study

**DOI:** 10.3389/fnhum.2025.1587153

**Published:** 2025-05-07

**Authors:** Xueyi Zhang, Qi Wang, Qiujie Li, Hui Liu, Xianglin Wan

**Affiliations:** ^1^School of Sport Science, Beijing Sport University, Beijing, China; ^2^Key Laboratory for Performance Training and Recovery of General Administration of Sport of China, Beijing, China; ^3^People’s Hospital of Queshan, Zhumadian, Henan, China; ^4^China Institute of Sport and Health Science, Beijing Sport University, Beijing, China

**Keywords:** stroke, dual-task, gait, variability of gait, dual-task cost

## Abstract

**Background:**

The application of dual-task walking paradigms for gait assessment in stroke patients is critical, where varying concurrent tasks may elicit distinct gait patterns of dual-task interference. This study assessed the acute effects of different types of dual tasks on gait in stroke patients during task performance, informing occupational, and physical therapists about care recommendations to prevent patients from falling and improve their balance function in daily life.

**Methods:**

A total of 19 stroke patients (52.7 ± 6.9 years old) performed the walking-only and dual-task walking (motor, arithmetic and speech) task test while a 3D motion capture system measured the gait parameters (the gait spatial-temporal parameters, sagittal angle of lower-limb joints, gait parameter variability and dual-task cost). One-way repeated measures ANOVA was used to test the effects of the above four walking conditions on gait parameters.

**Results:**

Arithmetic task and speech task interference can affect the gait of stroke patients (*P* < 0.05). Arithmetic task interference has the greatest impact on step speed, cadence, single support phase, hip joint range in support period and has the greatest dual-task cost, speech task interference has the greatest impact on cadence coefficient of variation (*P* < 0.05). The motor task was not significantly different from walking-only (*P* >0.05).

**Conclusion:**

Both arithmetic and speech tasks have a great impact on gait in stroke patients. Faced with cognitive interference, stroke patients spontaneously adopted a “cautious gait” walking pattern. In future rehabilitation training, diversity of task types is critical for gait rehabilitation training based on the walking ability of the patients.

## 1 Introduction

Stroke as a critical cause of death and disability in adults can lead to a decrease in walking and balance abilities, significantly impacting quality of life (Feigin et al., 2022). Therefore, gait assessment in stroke patients and increased attention to those at high risk of falls are essential. Dual-task walking refers to the simultaneous performance of walking and an additional concurrent task, which primarily includes cognitive-walking tasks and motor-walking tasks (Iqbal et al., 2020; Ramírez and Gutiérrez, 2021). Dual-task interference (DTI) arising from dual-task conditions is employed in clinical practice to assess walking ability and evaluate fall risk (Leone et al., 2017; Montero-Odasso et al., 2012).

Increased DTI may elevate the risk of falls in patients. Since stroke patients commonly experience cognitive and gait impairments, DTI further complicates the challenge of maintaining stable gait (Chisholm et al., 2014). Patients often exhibit characteristics such as reduced walking speed, gait variability, and increased dual task costs, which are strong predictors of increased fall risk (Muir-Hunter and Wittwer, 2016). The higher the difficulty level of gait training tasks, the greater the gait variability in stroke patients, leading to a higher risk of falls (Muir-Hunter and Wittwer, 2016; Thomson et al., 2020). Research indicates that stroke patients under dual task conditions may adopt a “cautious gait” walking pattern by reducing speed and stride length to prevent falls (Liu et al., 2018). Therefore, when implementing dual task paradigms in rehabilitation training, the complexity of tasks should be considered, and appropriate difficulty levels should be selected to match the capabilities of the target population (Chu et al., 2013).

Studies suggest that the complexity of dual tasks can be reflected in gait characteristics and is related to the type of tasks (Hausdorff et al., 2008). Holding a cup filled with water, sequential subtraction and verbal expression are close to the daily life of stroke patients, so they are often used as concurrent tasks in the research of dual-task walking paradigm (Al-Yahya et al., 2011). Compared to cognitive-motor dual tasks that are more relevant to stroke patients, healthy adults exhibit lower walking speeds under motor dual tasks (e.g., carrying a cup-walking) (Madehkhaksar and Egges, 2016). Among different cognitive tasks, the Stroop task has a greater impact on walking speed in healthy individuals compared to arithmetic tasks and reaction time tasks (Patel and Bhatt, 2014). However, current research cannot conclusively determine the differences in the impact of motor tasks compared to various types of cognitive tasks on gait, and whether different task types have varying effects on the risk of falls in stroke patients while walking remains unclear. Additionally, most analyses in existing studies on stroke patients’ gait performance focus on basic gait parameters such as walking speed and stride length, which may not fully reflect the patients’ walking stability and the extent of DTI exhibited under different types of dual tasks. The effects of different types of dual tasks on gait parameter variability and dual task costs in stroke patients are still unclear. Understanding the effects of different types of dual tasks on gait performance can inform occupational and physical therapists about care recommendations to prevent patients from falling and improve their balance function in daily life.

Therefore, this study aims to compare the immediate effects of motor, arithmetic, and speech dual tasks on the gait characteristics of stroke patients, to clarify the differences in the impact of different types of dual tasks on patients’ gait and provide a theoretical basis for developing dual task training programs to improve the walking abilities of stroke patients in the future. The study hypothesizes that different types of dual tasks have varying effects on stroke patients’ walking speed, stride length, lower limb joint angles, variability, and dual task costs, with the most significant impact from the arithmetic task among the three tasks.

## 2 Materials and methods

### 2.1 Study design

This cross-sectional study was conducted at the rehabilitation center of People’s Hospital of Queshan from April 2021 to May 2021. Participants were recruited through consecutive sampling.

### 2.2 Participants

The sample size was calculated using G*Power 3.1 software. Previous studies (Wrightson et al., 2016) report the large effects of dual-task walking performance. We adopted the same approach as Wrightson et al., using partial eta squared (η^2^ = 0.26) as the estimated effect size (Wrightson et al., 2020). 12 participants would be required to detect this effect (α = 0.05, β = 0.95) in a one-way repeated measure ANOVA determining a minimum of 12 stroke patients needed. Therefore, 19 stroke patients were recruited at the People’s Hospital of Queshan County, Henan Province, all experiencing their first episode within a year. General information of the participants is presented in Table 1.

**TABLE 1:** Participant demographics data (*N* = 19).

Gender		Paretic side		Stroke type		Age (years)	Post-stroke duration (months)	Height (cm)	Weight (Kg)	MMSE	FAC
Male	Female	Left	Right	Ischemic	Hemorrhagic						
15	4	13	6	12	7	52.7 ± 6.9	4.9 ± 2.1	166.7 ± 6.8	73.0 ± 6.9	25.9 ± 1.7	4.0 ± 0.6

MMSE, Mini-Mental State Examination; FAC, Functional Ambulation Category Scale.

Inclusion criteria: (1) aged 18 and above, (2) clinically diagnosed with a stroke and currently in the chronic phase, (3) able to hold a 200 ml water cup using the paretic limb and walk independently for 10 meters without any assistive devices, (4) clear consciousness and able to understand instructions. Patients with Mini-Mental State Examination (MMSE) scores greater than 24 were included (Folstein et al., 1975).

Exclusion criteria: (1) presence of other gait-affecting diseases, (2) unilateral neglect or other sensory impairments, (3) suffer from aphasia or other speech and language pathologies.

Patients refrained from vigorous exercise 24 h before the experiment and signed informed consent. The Functional Ambulation Category Scale (FAC) was used to assess walking ability in all enrolled participants. This study was approved by the Ethics Committee of Sports Science Experiments at Beijing Sport University (No: 202108OH).

### 2.3 Experimental procedure

The starting point and the endpoint were marked on the ground, approximately 10 meters apart. To exclude acceleration and deceleration phases, gait cycles were collected from a 5-m section starting 3 meters beyond the starting point. Before the formal test, participants were asked to walk on the test trail to familiarize themselves with the test site. During the test, the participants wore standardized tight-fitting clothing and their own sports shoes. Nineteen reflective markers were attached to the bilateral lower limbs of the participants according to the Helen-Hayes model (Zamporri and Aguinaldo, 2018). Each task required the participant to walk from the starting point to the endpoint. Initially, a walking-only task gait test was conducted where the participant walked on level ground at a comfortable pace. Subsequently, a dual task gait test involving motor/cognitive load was performed, with tasks such as motor (holding a cup of water), arithmetic, and speech tasks carried out in a random order. The task order was randomized using a random number generator to generate a sequence. Participants were assigned a number in the order of their enrollment and performed the tasks according to the sequence corresponding to their assigned number. During the motor-walking task, participants were required to hold a cup containing 150 mL of water (total capacity: 200 mL, without a lid) exclusively with their paretic side while walking, maintaining the water level as stable as possible without switching hands during the walking trial. In the arithmetic-walking task, the participant was instructed to subtract 7 from a randomly selected three-digit number between 100 and 199 (Zamporri and Aguinaldo, 2018). In the speech-walking task, the participant was asked to list as many words as possible from a specific category (e.g., countries, animals, fruits, vegetables, dishes, furniture; Yang et al., 2016). If an error occurred in the two cognitive-walking tasks, correction was not required, and the participant continued walking without interruption.

A valid trial was recorded when the participant successfully completed the motor/cognitive tasks and reached the endpoint. To minimize the effects of learning and participant fatigue, two valid data collections were obtained for each condition of gait testing (Oh-Park et al., 2013).

### 2.4 Data acquisition and processing

In this study, a 6-camera Qualisys Oqus 400 motion capture system (Qualisys, Gothenburg, Sweden) was employed to record the coordinates of surface markers on the paretic side. The markers’ coordinates were acquired at a sampling frequency of 200 Hz. To reduce measurement noise, the raw coordinate data were processed using a fourth-order Butterworth low-pass filter with a cut-off frequency of 13.3 Hz (Yu et al., 1999). A gait cycle was defined as the interval between successive heel strikes of the same foot during walking.

#### 2.4.1 Spatio-temporal gait parameters

Calculations were performed using the coordinates of the heel markers to determine gait speed, stride length, step width, cadence, and single support phase (Table 2).

**TABLE 2:** Definition of spatio-temporal gait parameters.

Parameters	Definition method
Step speed (cm/s)	The distance of anteroposterior distance per unit time in the paretic side
Step length (cm)	The anteroposterior distance between the non-paretic side’s heel strike and the next paretic side’s heel strike
Step width (cm)	The mediolateral distance between the non-paretic side’s heel strike and the next paretic side’s heel strike
Cadence (step/min)	Number of steps taken per minute
Single support phase (%)	The ratio of single support phase of the paretic side to the total duration of the gait cycle

#### 2.4.2 Lower limb joint angle

The hip joint center was determined using the Helen-Hayes lower limb landmark coordinates in accordance with the methodological framework established by Bell et al. (1989). The knee joint axis of rotation is the midpoint between the medial and lateral epicondyles of the femur, while the ankle joint axis of rotation is the midpoint between the medial and lateral malleoli. Joint Range of Motion (RoM) is defined as the difference between the maximum and minimum joint angles. This study computes and analyzes the angles of the paretic hip, knee, and ankle joints at the time of toe-off and heel-strike, as well as the sagittal plane RoM during the stance and swing phases of gait.

#### 2.4.3 Variability of gait parameters

Gait variability is widely employed to evaluate patients’ ability to adapt to gait-altering conditions and to assess fall risk (Chisholm et al., 2014). The coefficient of variation (CoV) was used to evaluate gait variability in stroke patients in this study (Radovanovic et al., 2020). For each participant, at least two complete gait cycles were recorded bilaterally in each trial. The four gait cycles (two per trial) from two trials were used to calculate the CoV for spatiotemporal gait parameters using the formula:


$$C\,o\,V=\frac{S\,D}{M\,e\,a\,n}\times 100\%$$

where SD and Mean represent the standard deviation and mean of a specific parameter, respectively.

#### 2.4.4 Dual-task cost

The dual-task cost (DTC) of participants walking was calculated using step speed, with the following formula (Doumas et al., 2009).


$$D\,T\,C=\frac{\left(S\,t\,e\,p\,S\,p\,e\,e\,d_{D\,u\,a\,l-t\,a\,s\,k}-S\,t\,e\,p\,S\,p\,e\,e\,d_{W\,a\,l\,k\,i\,n\,g-o\,n\,l\,y}\right)}{S\,t\,e\,p\,S\,p\,e\,e\,d_{W\,a\,l\,k\,i\,n\,g-o\,n\,l\,y}}\times 100\%$$

### 2.5 Statistical analysis

Statistical analysis was conducted using SPSS 23.0 software, and all data were expressed as mean ± standard deviation. All variables passed the Shapiro-Wilk normality test and Levene’s test for homogeneity of variances. A one-way repeated measures ANOVA was conducted to examine the effects of different types of tasks on gait parameters. *Post-hoc* pairwise comparisons with Bonferroni correction were conducted in SPSS 23.0, which directly outputs corrected *p*-values. Statistical significance was evaluated at the 0.05 level for the adjusted results. Hedge’s g was used as an index to evaluate the effect size.

## 3 Results

### 3.1 Spatio-temporal gait parameters

The ANOVA results (Table 3) revealed that different types of tasks have a significant impact on the step speed, step length, cadence, and single support phase of stroke patients (*P* < 0.05). Subsequent tests reveal that the step speed during arithmetic tasks is lower than during motor tasks (MD = –14.75, *P* = 0.002, Hedge’s *g* = 0.61) and walking-only (MD = –14.54, *P* = 0.003, Hedge’s *g* = 0.57). The step speed during speech tasks being lower than during motor tasks (MD = –8.95, *P* = 0.014, Hedge’s *g* = 0.35). The step length during arithmetic tasks is not significantly different from speech tasks (*P* = 0.156), but both are lower than during motor tasks (MD = –5.83, *P* < 0.001, Hedge’s *g* = 0.40; MD = –3.67, *P* = 0.040, Hedge’s *g* = 0.25) and walking-only (MD = –6.19, *P* < 0.001, Hedge’s *g* = 0.40; MD = –4.03, *P* = 0.039, Hedge’s *g* = 0.25). The cadence during arithmetic tasks is lower than during motor tasks (MD = –11.32, *P* = 0.017, Hedge’s *g* = 0.59) and walking-only (MD = –10.44, *P* = 0.019, Hedge’s *g* = 0.54). The single support phase during arithmetic tasks is lower than during speech tasks (MD = –1.68, *P* = 0.038, Hedge’s *g* = 0.18), motor tasks (MD = –3.30, *P* = 0.007, Hedge’s *g* = 0.38), and walking-only (MD = –3.33, *P* = 0.006, Hedge’s *g* = 0.37).

**TABLE 3:** Spatio-temporal gait parameters under four task types.

Parameters	Walking-only	Motor task	Arithmetic task	Speech task	*P*-value	*F*-value	Partial η ^2^
Step speed (cm/s)	55.0 ± 28.3	55.2 ± 26.1	40.5 ± 20.9^ab^	46.3 ± 23.6^b^	0.007	6.05	0.57
Step length (cm)	42.3 ± 16.1	42.0 ± 14.7	36.1 ± 13.9^ab^	38.3 ± 14.5^ab^	<0.001	14.61	0.48
Step width (cm)	18.0 ± 8.1	18.2 ± 8.6	18.7 ± 8.2	19.0 ± 8.9	0.314	1.22	0.07
Cadence (step/min)	75.0 ± 19.2	75.9 ± 18.8	64.6 ± 18.3^ab^	69.5 ± 19.6	0.032	3.92	0.46
Single support phase (%)	23.6 ± 8.6	23.6 ± 8.1	20.3 ± 8.8^abc^	22.0 ± 9.4	0.005	6.76	0.59

a, b, and c represent significant differences compared with walking-only, motor task, and arithmetic task, respectively.

### 3.2 Lower limb joint angle

The ANOVA results (Table 4) revealed that different types of tasks have a significant impact on the RoM of hip joint support phase, hip joint swing phase, and knee joint swing phase in stroke patients (*P* <0.05). Subsequent tests reveal that the hip joint support phase RoM during arithmetic tasks is lower than during motor tasks (MD = –3.83, *P* = 0.012, Hedge’s *g* = 0.39) and walking-only (MD = –4.34, *P* = 0.003, Hedge’s *g* = 0.44). The hip joint swing phase RoM during arithmetic tasks is lower than during motor tasks (MD = –2.37, *P* = 0.017, Hedge’s *g* = 0.34). The knee joint swing phase RoM during speech tasks is lower than during motor tasks (MD = –5.76, *P* = 0.024, Hedge’s *g* = 0.30) but not significantly different from walking-only (*P* = 0.232).

**TABLE 4:** Paretic side lower limb joint angle under four task types (°).

Joint	Parameters	Walking-only	Motor task	Arithmetic task	Speech task	*P*-value	*F*-value	Partial η ^2^
Hip	Heel strike angle	4.1 ± 11.2	1.9 ± 13.2	1.7 ± 12.2	2.1 ± 9.6	0.770	0.10	0.02
	Toe off angle	1.2 ± 10.0	1.2 ± 10.8	0.6 ± 9.7	1.9 ± 10.4	0.841	0.32	0.02
	Mean support phase RoM	29.6 ± 9.4	29.1 ± 9.3	25.3 ± 9.7^ab^	26.8 ± 9.6	<0.001	10.45	0.40
	Mean swing phase RoM	16.4 ± 7.3	17.0 ± 7.2	14.6 ± 6.7^b^	15.1 ± 7.1	0.034	3.82	0.45
Knee	Heel strike angle	16.7 ± 7.8	16.9 ± 8.5	14.2 ± 4.8	16.7 ± 8.3	0.236	2.26	0.13
	Toe off angle	33.4 ± 14.5	34.8 ± 14.4	30.2 ± 13.5	33.3 ± 13.5	0.623	0.80	0.04
	Mean support phase RoM	40.5 ± 16.7	39.6 ± 16.1	37.4 ± 15.1	36.4 ± 15.5	0.065	2.87	0.05
	Mean swing phase RoM	39.1 ± 19.5	39.4 ± 19.2	35.7 ± 16.5	33.7 ± 17.6^b^	0.026	4.21	0.47
Ankle	Heel strike angle	7.2 ± 10.1	6.4 ± 10.4	7.4 ± 14.1	6.4 ± 11.2	0.869	0.08	0.01
	Toe off angle	10.2 ± 8.5	8.2 ± 10.7	8.8 ± 12.5	8.1 ± 9.9	0.592	0.50	0.12
	Mean support phase RoM	20.4 ± 5.9	21.0 ± 6.7	19.0 ± 5.3	20.2 ± 6.4	0.102	2.19	0.12
	Mean swing phase RoM	10.4 ± 4.0	10.0 ± 4.6	9.7 ± 4.1	8.9 ± 4.0	0.328	1.18	0.07

a and b represent significant differences compared with walking-only, motor task, and arithmetic task, respectively. RoM, range of motion.

### 3.3 Variability of gait parameters

The ANOVA results (Table 5) revealed that different types of tasks have a significant impact on the CoV of gait speed, step length, and cadence in stroke patients (*P* < 0.05). Subsequent tests indicate that the CoV of step speed in the arithmetic task is higher than in the motor task (MD = 5.66, *P* = 0.033, Hedge’s *g* = 0.69). The CoV of step length in the speech task is higher than in the motor task (MD = 3.68, *P* = 0.037, Hedge’s *g* = 0.69). The CoV of cadence in the speech task is higher than in the motor task and walking-only task (MD = 2.86, *P* = 0.050, Hedge’s *g* = 0.83; MD = 1.98, *P* = 0.041, Hedge’s *g* = 0.45).

**TABLE 5:** CoV of gait parameters under four task types (%).

Parameters	Walking-only	Motor task	Arithmetic task	Speech task	*P*-value	*F*-value	Partial η ^2^
Step speed CoV	7.1 ± 4.9	5.7 ± 2.9	11.4 ± 6.9^b^	11.0 ± 9.4	0.009	4.31	0.21
Step length CoV	5.7 ± 5.7	4.3 ± 2.8	7.3 ± 4.4	8.0 ± 6.8^b^	0.035	3.12	0.16
Step width CoV	10.3 ± 8.0	8.5 ± 8.4	8.8 ± 9.1	8.8 ± 8.2	0.625	0.60	0.11
Cadence CoV	4.6 ± 4.1	3.7 ± 2.0	6.8 ± 3.5	6.5 ± 4.2^ab^	0.010	4.20	0.21
Single support phase CoV	5.2 ± 3.4	5.3 ± 3.5	9.8 ± 9.8	7.6 ± 7.0	0.397	1.06	0.19

a and b represent significant differences compared with walking-only, motor task, and arithmetic task, respectively. CoV, coefficient of variation.

### 3.4 Dual-task cost

The ANOVA results (Table 6) revealed that different types of tasks have a significant impact on DTC (*P* < 0.001). Subsequent tests demonstrated that arithmetic task is higher than in the motor task and walking-only task (MD = 26.36, *P* = 0.001, Hedge’s *g* = 1.41; MD = 22.73, *P* = 0.003, Hedge’s *g* = 1.48). The DTC in the speech task is higher than in the motor task (MD = 17.23, *P* = 0.004, Hedge’s *g* = 0.95).

**TABLE 6:** DTC under four task types (%).

Parameters	Walking-only	Motor task	Arithmetic task	Speech task	*P*-value	*F*-value	Partial η ^2^
DTC	0	3.6 ± 14.5	-22.7 ± 21.2^ab^	-13.6 ± 20.4^b^	<0.001	13.75	0.46

a and b represent significant differences compared with walking-only, motor task, and arithmetic task, respectively. DTC, dual-task cost.

## 4 Discussion

The study findings provide partial support for the hypothesis. Arithmetic tasks significantly reduced gait speed, cadence, single support phase duration, and hip RoM during the support phase, while increasing DTC. Speech tasks most severely impaired dynamic stability, with marked increases in step length CoV and cadence CoV, alongside reduced knee RoM during the swing phase. Motor tasks exhibited no statistically significant differences compared to walking-only across all measured parameters.

Carrying a cup filled with water while walking as a dual task does not significantly affect the gait of stroke patients, which may be related to the attention control patterns of stroke patients. Previous studies have shown that motor-walking dual task significantly decreases the gait speed of healthy adults (Madehkhaksar and Egges, 2016), but in this study, the motor task did not affect the gait speed of stroke patients. This may be because healthy individuals typically walk in an automated mode during single-task walking, resulting in faster gait speeds. When faced with dual task conditions, they reduce their gait speed and gait variability to compensate for the increased task challenges during dual task walking (Kao et al., 2015). However, stroke patients, due to brain damage leading to insufficient attention resources and reduced flexibility in attention allocation (Plummer et al., 2020; Yang et al., 2018), exhibit characteristics such as unstable posture and muscle stiffness, representing an inefficient and conscious attention control pattern (Dehmiyani et al., 2022). Therefore, stroke patients already require high attention control during single-task walking, resulting in slower gait speeds. Even though adding a motor task requires increased brain activity to maintain motor performance (Chen et al., 2022), they do not show differences in gait compared to single-task walking. Moreover, the allocation of attention resources during dual task walking may be achieved through synchronization and coordination of relevant brain areas such as the prefrontal cortex, premotor cortex, and supplementary motor area (Chen et al., 2022). Studies have found that in Parkinson’s patients performing the cup-water-walking task, there is increased activation in the early stages of the supplementary motor area and premotor cortex, but this activation does not persist in the later stages of the task (Liu et al., 2022). This may be because carrying the cup of water involves a fixed action that does not require sustained high attention, thus not significantly interfering with gait. However, Coh et al. found that stroke patients exhibited slower gait speeds during water-carrying walking tasks compared to walking-only (Goh et al., 2017), which contrasts with our findings. This discrepancy may be attributed to the smaller cup capacity (200 mL) and the remaining distance between the water surface and the cup rim in our study, which reduced the physical burden on participants. Stroke patients tend to exhibit greater cognitive-motor interference in more complex motor tasks (Rice et al., 2022), yet in this study, the motor task DTC does not significantly differ from walking-only, indicating that the motor task designed in this study has relatively low cognitive task difficulty and does not place high demands on patients’ attention resources during balance and walking. Therefore, when using motor dual tasks for rehabilitation training, it is important to incorporate varied motor tasks such as walking while bouncing a rubber ball to avoid diminishing the effectiveness of rehabilitation training.

Comparing motor tasks, arithmetic tasks and speech tasks increase the risk of falls in stroke patients during walking. Gait variability is commonly used to assess fall risk, determine patients’ ability to adapt to gait changes (Muir-Hunter and Wittwer, 2016), and increasing gait variability can reflect an increase in task difficulty. Higher gait variability is associated with impaired sensory-motor control, leading to increased energy expenditure and decreased dynamic balance during walking (Kim et al., 2021). Increased step length variability may indicate poor control of forward progression (Chisholm et al., 2014), reflecting degradation in muscle and proprioceptive control functions (Shaffer and Harrison, 2007), thereby increasing the risk of falls in stroke patients during walking. The extent of gait changes is related to secondary task demands, tasks requiring more cognitive functions lead to higher DTC in walking tasks (Lu et al., 2015). CMI in dual tasks is negativel correlated with gait speed, and when speed DTC ≥ 20%, the level of CMI is considered high (Baek et al., 2021). In this study, stroke patients showed decreased step speed and DTC exceeding 20% during cognitive tasks, indicating a high CMI gait pattern and potentially increased fall risk. Therefore, in the early stages of rehabilitation training, it is advisable to reduce the difficulty of cognitive and speech tasks to lower the risk of falls in stroke patients.

Additional cognitive tasks increase the risk of falls in stroke patients, leading them to adopt a cautious gait pattern with reduced speed, stride length and hip RoM to prevent falls. This cautious gait pattern, driven by fear of falling, involves a protective gait mode with decreased speed, step length, and step width, paradoxically increasing the risk of falls (Bueno et al., 2019). The generation of this gait pattern may be related to task priorities; in obstacle-free walking, stroke patients prioritize cognitive tasks at the expense of speed, while facing obstacles, they prioritize gait performance (Timmermans et al., 2018). This kinematic adaptation was further evidenced by reduced hip RoM, as participants exhibited restricted stride length through deliberate limitation of hip excursion, particularly during arithmetic tasks. The findings of previous studies involving healthy individuals have demonstrated that alterations in lower limb joints during dual-task walking are predominantly observed in the hip and knee joints, which aligns with the observations made in the present investigation (Vandenheever and Lambrechts, 2023). In this study designed for obstacle-free walking, stroke patients may ensure cognitive task completion by adopting a cognitive-priority walking strategy, compromising gait performance. Hence, in dual task training, without guiding the task priorities of stroke patients, they may develop a habit of cautious walking, affecting gait rehabilitation outcomes. The superiority of variable priority training over fixed priority training in dual tasks is still debated (Muehlbauer et al., 2022), requiring further research for clarification.

Arithmetic tasks and speech tasks have different impacts on the gait of stroke patients. Different cognitive tasks may have varying effects on stroke patients’ gait possibly due to their different attention control mechanisms. Serial arithmetic relies on working memory (Hittmair-Delazer et al., 1994), a system for temporary storage and processing of information directly related to executive functions. Combining calculation tasks with walking affects stroke patients’ executive functions, reducing their dynamic balance control level, allowing better handling of calculation tasks by lowering speed and single support phase duration for walking stability. Speech fluency relies on semantic memory (Weiss et al., 2003), and due to impaired semantic memory from brain damage in stroke patients, they exhibit increased stride length variability during walking. Current brain activation studies on dual tasks found that additional cognitive tasks during walking increase activation in the prefrontal cortex, premotor cortex, and posterior parietal cortex (Lim et al., 2022; Rahman et al., 2021), but it is unknown if different types of cognitive task interference lead to different activation areas or varying levels of activation. Different types of cognitive tasks have varied effects on patients, so when designing dual task rehabilitation programs, using a variety of tasks to stimulate different aspects of patients may yield better intervention effects compared to using a single cognitive task.

Due to technical constraints related to experimental environment, this study did not include a healthy control group and did not examine the impacts of cognitive load or motor task complexity on gait patterns in stroke patients. Future work should integrate tools like the Correct Cognitive Response metric (Penati et al., 2020) to concurrently evaluate cognitive and motor performance. Future studies could expand the participant pool to further investigate the differences in gait effects of dual task condition on stroke patients at different stroke types or stages of rehabilitation. Future studies could conduct prospective follow-ups on stroke patients, recording actual fall incidents to determine the relationship between performance under dual-task conditions and actual falls.

## 5 Conclusion

In the dual tasks selected in this study, the task of holding a cup of water did not significantly affect the gait of stroke patients. The arithmetic task and the speech task affect different aspects of the gait of stroke patients, both increasing the risk of falls. Faced with cognitive task interference, stroke patients spontaneously adopt a “cautious gait” walking pattern. In future rehabilitation training, it is necessary to choose motor tasks which is more variable, increase the types of dual task training, and select dual tasks of different difficulty levels based on the patient’s walking ability for gait rehabilitation training.

## Data Availability

The original contributions presented in the study are included in the article, further inquiries can be directed to the corresponding author.
